# Effects of shear emulsifying/ball milling/autoclave modification on structure, physicochemical properties, phenolic compounds, and antioxidant capacity of lotus (*Nelumbo*) leaves dietary fiber

**DOI:** 10.3389/fnut.2023.1064662

**Published:** 2023-02-23

**Authors:** Hui Zheng, Yan Sun, Tao Zheng, Yiqiong Zeng, Liping Fu, Tingting Zhou, Fan Jia, Yao Xu, Kai He, Yong Yang

**Affiliations:** ^1^College of Pharmacy, Hunan University of Chinese Medicine, Changsha, China; ^2^School of Pharmaceutical Science, Hunan University of Medicine, Huaihua, China

**Keywords:** lotus (*Nelumbo*) leaves, dietary fiber, shear emulsifying, ball milling, physicochemical properties, phenolic compounds

## Abstract

Lotus *(Nelumbo*) leaves are rich in polyphenols and dietary fiber, which have the potential as a high-quality fiber material in functional food. However, lotus leaves exhibit dense structure and poor taste, it is vital to develop appropriate modification methods to improve the properties of lotus leaves dietary fiber. In this study, the effects of three modification methods with shear emulsifying (SE), ball milling (BM), and autoclave treatment (AT) on structure, physicochemical properties, phenolic compounds, and antioxidant capacity of lotus leave dietary fiber (LDF) were evaluated. SEM indicated that there were significant differences in the microstructure of modified LDFs. FT-IR spectra and X-ray diffraction pattern of modified LDFs revealed similar shapes, while the peak intensity and crystalline region changed by modification. SE showed the greatest effect on crystallization index. SE-LDF had the highest water holding capacity, water swelling capacity, and bound phenolic content in LDFs, which increased by 15.69, 12.02, and 31.81%, respectively, compared with the unmodified LDF. BM exhibited the most dramatic effect on particle size. BM-LDF had the highest free phenolic and total phenolic contents in LDFs, which increased by 32.20 and 29.05% respectively, compared with the unmodified LDF. Phenolic compounds in LDFs were mainly free phenolic, and modifications altered the concents of flavonoids. The BM-LDF and SE-LDF exhibited higher antioxidant capacity than that of AT-LDF. Overall, SE-LDF showed better physical properties, and BM-LDF showed better bioactive components. SE and BM were considered to be appropriate modification methods to enhance the properties of LDF with their own advantages.

## 1. Introduction

Dietary fibers mean carbohydrate polymers with 3 or more monomeric units, which are resistant to the endogenous digestive enzymes and thus neither hydrolyzed nor absorbed in the small intestine of humans (1). They have shown a positive impact on human health, such as reducing blood sugar and blood lipids, preventing obesity, and regulating intestinal flora (1, 2), therefore they have become a functional food raw material favored broadly by the consumer in recent years. Dietary fibers extracted from plants are often combined with natural plant polyphenols which show antioxidant activity (3), and which exhibit better functional characteristics for to human health than pure dietary fibers (4). These dietary fibers can be obtained from agricultural residue, and reasonable exploitation can greatly improve the added value of agricultural products, which has attracted the attention of researchers (5). However many dietary fibers in plant sources show disadvantages such as compact structure and poor taste, which lead to their low exploitation and utilization, it is necessary to modify dietary fibers in plant sources. Many studies have found that appropriate modifications, such as high pressure, high heat, shearing, extrusion and cellulase hydrolysis, can improve the positive impact of dietary fibers on human health by changing the structure, chemical components, physicochemical and functional properties (3, 6–8). Therefore, it is of great importance to select appropriate modification methods of dietary fibers, which is directly related to their exploitation value.

Shear emulsifying (SE) is based on the high-speed rotation rotor that creates a strong fluid shear force between the inner rotor and external stator, meanwhile the high rotational speed also produces intense laminar, turbulent and cavitation effects(9). These comprehensive effects change the structure, conformation and several properties of samples (6). SE has been widely used in the food industry for emulsification, dispersion, chemical reactions, cell disruption, deagglomeration, etc (9). Many studies have shown that SE can improve the extraction rate of soluble dietary fiber in raw materials, such as bamboo shoot fiber (10), defatted walnut flour (11) and akebia trifoliata koidz. seeds (12), while there are few studies on the application of shear emulsifying on insoluble dietary fiber. Our previous studies preliminarily showed that SE significantly enhanced the water-holding capacity and expansibility of insoluble dietary fiber compared with other fiber modification treatments, so SE may have its unique advantages in the modification of insoluble dietary fiber. Ball milling (BM) is a technique that uses mechanical as well as thermal effects to change the physical and chemical properties of raw materials (7), which has become a common fiber modification method in the food industry. The samples of planetary ball milling are mainly ground by the pressure, collision, and absorption between the grinding balls and the inner wall of the jar(s). The temperature may rise at the same time in this process, resulting in thermal effects (13). Due to these effects, BM can rapidly reduce the particle size of dietary fibers and modify the various properties of the material, such as asparagus leaf (3), grape pomace (13), soybean insoluble dietary fiber (14) and sea buckthorn insoluble dietary fiber (15). Autoclave treatment (AT) is a frequent method of food processing and sterilization. The combination of pressure treatment and thermal treatment can destroy the structure of macromolecules and lead to changes in functional properties, such as affecting the physical and chemical functions of fiber materials, affecting the dissolution rate of free polyphenols and antioxidant performance, which have been reported in many research reports, such as brewers' spent grain (8), whole grain oats (16), black bean (17), and waste orange peels (18).

Lotus (*Nelumbo*) originates in Asia and has seen cultivation for more than 3000 years as a food-stuff and a medicinal crop (19). The ancient literature of traditional Chinese medicine recorded lotus leaves can remove heart-fire and heart-heat, cool blood and arrest bleeding (20), and modern medical research has highlighted the promising health activities of lotus leaves including antioxidant activity, anti-diabetes, anti-obesity, anti-neurotic, anti-inflammation, anti-cancer, liver protection, etc (21, 22). Lotus *(Nelumbo*) leaves are rich in polyphenols compounds and dietary fiber (21), which meet the conditions as a source of high-quality dietary fibers. Therefore, lotus (*Nelumbo*) leaves dietary fiber (LDF) have a good exploitation potential which can be used as a high-fiber supplement in food processing. In recent years, there have been many studies on lotus *(Nelumbo*) leaves polyphenols (20, 22, 23), while the fiber components with large content in lotus *(Nelumbo*) leaves have hardly been reported. Due to the dense structure and poor taste of lotus *(Nelumbo*) leaves, it is vital to choose appropriate fiber modification methods to improve the exploitation value of lotus *(Nelumbo*) leaves. In this study, the effects of three modification methods with SE, BM, and AT on structure, physicochemical properties, phenolic compounds and antioxidant capacity of LDF were evaluated. The results of this research could providing an appropriate basis for further exploitation of LDF as a new high-quality fiber supplement in functional food.

## 2. Materials and methods

### 2.1. Materials

Dry lotus *(Nelumbo*) leaves was provided by Hunan Zhenxing Traditional Chinese Medicine Co., Ltd. (Changsha, Hunan, China). Cellulase (CAS 9012-54-8, S10041) was bought from Shanghai Yuanye Biotechnology Co., Ltd (Shanghai, China). Total dietary fiber assay kit (TDF-200A) was bought from Megazyme International Ireland Ltd (Bray, Ireland). Rutin, hyperoside, isoquercitrin, astragalin and quercetin were bought from Chengdu Aifa Biotechnology Co., Ltd (Chengdu, Sichuan, China). Catechin, myricetin and kaempferol were bought from Hefei Bome Biotechnology Co. Ltd (Hefei, Anhui, China). DPPH and ABTS were brought from Shanghai Maclean Biochemical Technology Co. Ltd (Shanghai, China). The rest of reagents were brought from Sinopharm Chemical Reagent Co., Ltd (Shanghai, China) and were analytical pure.

### 2.2. Modification treatment of lotus leaves powder

Dry lotus leaves, 4–7 cm long and 1–2 cm wide, were crushed with a pulverizer (FW-200, Beijing Zhongxing Weiye Instrument Co., Ltd, Beijing, China), and then passed through a 50-mesh sieve to obtain lotus leaves powder.

#### 2.2.1. SE modification

Lotus leafes powder (30 g) was mixed with 750 mL distilled water, and was sheared at 8000 rpm for 30 min with a shear emulsifying (FM20-D, Shanghai Fluko Technology Development Co., Ltd., Shanghai, China). The mixture was centrifuged at 5000 rpm for 15 min to remove the supernatant. The SE modified lotus leaves powder was obtained by freeze-drying and sealed until use.

#### 2.2.2. BM modification

Lotus leaves powder (50 g) was processed with a planetary ball mill (XQM-4, Changsha Tianchuang Powder Technology Co., Ltd, Changsha, Hunan, China), and mixed with 0.9 kilograms of zirconia balls in a 1-L vessel at 500 rpm for 30 min. The BM modified lotus leaves powder was obtained and sealed until use.

#### 2.2.3. AT modification

Lotus leaves powder (250 g) was spread over a stainless steel dish, added 50 mL of distilled water and stirred well, and placed in a 4°C refrigerator for 3 h. Then the stainless steel dish was placed in an autoclave (LDZX-5DKBS, Shanghai Shen an Medical Instrument Factory, Shanghai, China) for 30 min at 121°C (0.10 MPa). The AT modified lotus leaves powder was obtained by freeze-drying and sealed until use.

### 2.3. Lotus leaves dietary fiber preparation

Lotus leaves powder (100 g) was mixed with 1500 mL of hydrochloric acid at the pH 4.0 with 2.50 g cellulase addition. The mixture was incubated at 50°C, 250 rpm for 2 h with vibration, and was centrifugated at 5000 rpm for 20 min to remove the supernatant. The precipitate was then gathered and dried by freeze-drying to obtain LDF. The lotus leaves powder, SE modified lotus leaves powder, BM modified lotus leaves powder and AT modified lotus leaves powder were extracted to obtain control (unmodified LDF), SE-LDF, BM-LDF and AT-LDF, respectively.

### 2.4. Structure

#### 2.4.1. Scanning electron microscopy

The method was slightly modified according to Zhu et al. (15). The electron microscopy observation and photographing of sample was carried out using an SEM (EVO18, Carl Zeiss AG., Germany). Sample was loaded on a sample holder with double-sided conducting adhesive tapes, and coated with a gold layer. Subsequently, sample was observed at 10k × and 2k× magnification at 30.0 kv.

#### 2.4.2. Fourier transform infrared spectroscopy

The method was slightly modified according to Jiang et al. (12). FTIR spectra of samples were analyzed with an FTIR spectroscopy instrument (Nicolet 380, Thermo Fisher Scientific Inc, USA). Sample (2 mg) was mixed with 200 mg of KBr and then pressed into one slice. FITR spectra was recorded in the full wavelength of 4000–400 cm^−1^. The mixture was scanned for 32 times at a resolution of 4 cm^−1^.

#### 2.4.3. X-ray diffraction

XRD analysis of sample was carried out as described by Zheng et al. (24) and measured by an X-ray diffractometer (D8 Advance, Brooke AXS Co., Ltd., Germany). The determination was done at room temperature using Cu-Kα radiation source with a step size of 0.02°. The diffraction angle (2θ) was performed from 5 to 50° with a speed of 1°/min. The crystallinity index (CrI) of sample was calculated following equation.


(1) $$CrI=\frac{I_{002}-I_{am}}{I_{002}}\times 100$$

where *I*_002_ is the intensity of the 002 lattice diffraction at 2θ = 22°, and *I*_am_ is the intensity of diffraction at 2θ = 18°. *I*_am_ represents the amorphous region (amorphous cellulose, hemicellulose and lignin).

### 2.5. Physical and chemical analysis

#### 2.5.1. Dietary fiber content determination

Soluble dietary fiber (SDF), insoluble dietary fiber (IDF) and total dietary fiber (TDF) of sample were measured according to AOAC 991.43 using a total dietary fiber assay kit.

#### 2.5.2. Particle size determination

The method was slightly modified according to Chitrakar et al. (3). Particle size of sample was measured by a laser particle size analyzer (Bettersize2600E, Dandong Better Instrument Co., Ltd., Dandong, Liaoning, China). Distilled water was selected as the dispersant. D_10_, D_50_, and D_90_ are equivalent volume diameters determined at 10, 50, and 90% cumulative volume, respectively. A_sf_ represents specific surface areas of powder.

#### 2.5.3. Water-holding capacity

The method was slightly modified according to Luo et al. (25). Sample (0.5 g) was well mixed with 10 mL of distilled water in a centrifuge tube at room temperature for 18 h. The supernatant was removed by centrifugation at 4000 rpm for 15 min, and the residue was immediately collected and weighted. WHC was calculated according to the following equation.


(2) $$WHC(g/g)=\frac{w_{1}-w}{w}\;$$

Where *w* is the weight of dried sample (g), and *w*_1_ is the weight of the residue (g) containing water.

#### 2.5.4. Oil-holding capacity

The method was slightly modified according to Luo et al. (25). Sample (0.5 g) was well mixed with 10 mL of peanut oil in a centrifuge tube at room temperature for 18 h. The supernatant was removed by centrifugation at 4000 rpm for 15 min, and the residue was immediately collected and weighted. OHC was calculated according to the following equation.


(3) $$OHC(g/g)=\frac{m_{1}-m}{m}$$

Where *m* is the weight of dried sample (g), and *m*_1_ is the weight of the residue (g) containing oil.

#### 2.5.5. Water swelling capacity

The method was slightly modified according to Wang et al. (26). Sample (0.5 g) was placed in a test tube, 5 mL of water was added and it was hydrated for 18 h at 4°C. WSC was calculated according to the following equation.


(4) $$WSC(mL/g)=\frac{V_{1}-V}{m}$$

Where *V* is the volume of dried sample (mL), *V*_1_ is the volume of the hydrated sample (mL), and *m* is the weight of dried sample (g).

#### 2.5.6. Color

The method was slightly modified according to Chen et al. (14). The color of sample was measured by a colorimeter analyzer (CS-820N, Hangzhou Caipu technology co., ltd., Hangzhou, Zhejiang, China). The color value was represented by the CEL L^*^, a^*^, b^*^ values, where L^*^ represents the brightness (0 is black, 100 is white); a^*^ represents the redness and greenness (a^*^ > 0, represents the degree of red; a^*^ < 0 means green degree); b^*^ means yellow and blue degree (b^*^ > 0 means yellow degree, b^*^ < 0 means blue degree). Color difference (ΔE) was calculated using the following equation. L_0_, a_0_, and b_0_ represent the colors of control, which used for comparison.


(5) $$\Delta E=\sqrt{{\left(L^{*}-L_{0}^{*}\right)}^{2}+{\left(a^{*}-a_{0}^{*}\right)}^{2}+{\left(b^{*}-b_{0}^{*}\right)}^{2}}\;$$

### 2.6. Polyphenol extraction and determination

#### 2.6.1. Extraction of phenolic compounds

Extraction of free phenolic (FP) was slightly modified according to Sameera et al. (27). Sample (1 g) was mixed with 25 mL of acetone/methanol/water (40:40:20, *v/v/v*). The mixture was placed in a shaking incubator at 40°C, at 250 rpm for 1 h and centrifuged at 5 000 rpm for 10 min. After three repeat extractions, the supernatants were pooled and the solvent was rotary evaporated in vacuo at 40°C to obtain FP extract. The solids remaining after the extraction was used for the extraction of bound phenolic (BP).

Extraction of BP was slightly modified according to Dong et al. (28). The solids remaining after the extraction of FP was mixed with 10 mL of 2 M sodium hydroxide. The mixture was placed in a shaking incubator at 250 rpm for 3 h at room temperature, then acidified with 6 M hydrochloric acid to pH 2 and centrifuged at 5000 rpm for 10 min. The supernatant was extracted with ethyl acetate, and the extraction was repeated three times. The ethyl acetate phase was collected, and the solvent was rotary evaporated in vacuo at 40°C to obtain BP extract.

#### 2.6.2. Polyphenol content determination

Polyphenol contents of FP and BP extracts were determined using the Folin-Ciocalteu method according Tian et al. (29), with slight modifications. Extract solution (100 μL) was mixed with 7.9 mL of distilled water and 500 μL of Folin-Ciocalteu reagent, then after 5 min, 1.5 mL of sodium carbonate solution (20%, *w/v*) was added. After resting for 2 h at room temperature, in the dark, the absorbance at 765 nm was measured using a spectrophotometer (UV-1780, Suzhou Shimadzu Instrument Co., Ltd., Suzhou, Jiangsu, China). The result was expressed as mg of gallic acid (GA) equivalent in 1 g dry weight of sample (mg GA eq/g DW).

#### 2.6.3. Flavonoids composition determination by HPLC

The method was slightly modified according to Chen et al. (19). The flavonoids composition in different samples was determined by HPLC (1260, Agilent Technologies Co. Ltd., USA) with a reversal phase column (XB-C18, Agilent Technologies Co. Ltd., USA). FP and BP extract solution were filtered through a 0.45 μm microporous membrane before injection. HPLC conditions: column temperature 30°C, detection wavelength 360 nm, injection volume 5 μL, flow rate 0.8 mL/min. The mobile phases were acetonitrile (A) and formic acid/water (0.1:99.9, *v/v*). The program of gradient elution: 0–10 min, 15% A; 10–25 min, 15–20% A; 25–40 min, 20–25% A; 40–55 min, 25–30% A; 55–60 min, 30–25% A; 60–70 min, 25–15% A.

### 2.7. Antioxidant capacity

#### 2.7.1. DPPH free radical scavenging capacity

The method was slightly modified according to Ye et al. (30). FP and BP extract solution (100 μL) was mixed with 3.5 mL of DPPH solution (0.1 mmol/L in methanol) and left to stand for 20 min in the dark, then the absorbance was measured at 517 nm by a UV Visible Spectrophotometer (UV-1780, Suzhou Shimadzu Instrument Co., Ltd., Suzhou, Jiangsu, China). The result was expressed in micromoles of Trolox equivalent to 1 g dry weight of sample (μmol Trolox eq/g DW).

#### 2.7.2. ABTS free radical scavening capacity

The method was slightly modified according to Wootton-Beard et al. (31). 7.0 mmol/L ABTS solution (400 mL) was mixed with 200 mL of 2.45 mmol/L potassium persulfate, and let stand for 12–16 h in the dark to form stable stock ABTS^+^· solution. The ABTS^+^· stock solution was diluted with deionized water to an absorbance of 0.70 ± 0.02 at 734 nm wavelength for later using. 50 μL of FP and BP extract solution was mixed with 4.9 mL of ABTS^+^· solution and left to stand for 10 min in a dark environment at room temperature, then measured the absorbance at a wavelength of 734 nm by a UV Visible Spectrophotometer (UV-1780, Suzhou Shimadzu Instrument Co., Ltd., Suzhou, Jiangsu, China). The result was expressed in micromoles of Trolox equivalent to 1 g dry weight of sample (μmol Trolox eq/g DW).

### 2.8. Statistics analysis

All samples were prepared and analyzed in triplicate, and the experimental results are expressed as the mean ± standard deviation (Mean ± SD). The statiscal analyses were performed with IBM S*P*SS Statistics 23.0. One-way ANOVA was applied, followed by Duncan's multiple range test for mean comparisons and difference significance analysis (*p* < 0.05).

## 3. Results and discussion

### 3.1. Structural characterization

#### 3.1.1. Scanning electron microscopy

**Figure 1:**
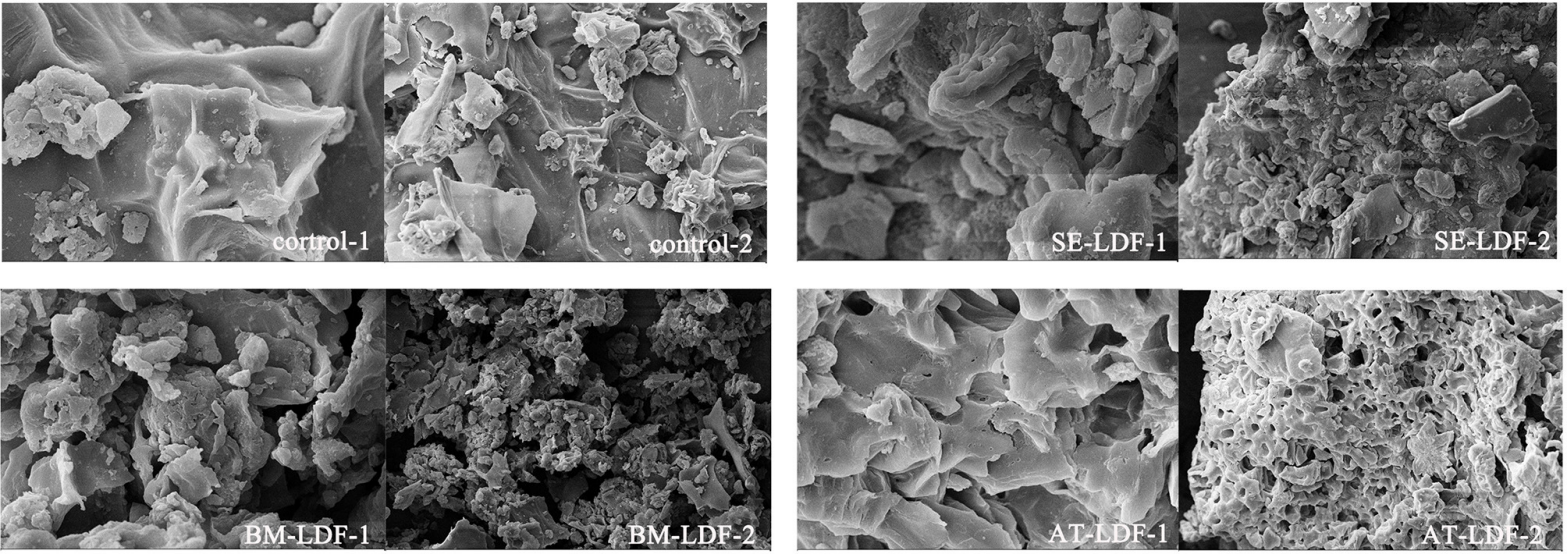
SEM images of control, SE-LDF, BM-LDF, and AT-LDF. Magnification: 1 (10 k ×), 2 (2 k ×). Control: lotus leaves dietary fiber. SE-LDF, shear emulsifying modified lotus leaves dietary fiber; BM-LDF, ball milling modified lotus leaves dietary fiber; AT-LDF, autoclave modified lotus leaves dietary fiber.

The differences in many physicochemical properties of dietary fibers can be explained by the microstructure, such as dissolving out the capacity of functional components, adsorption capacity, water swelling capacity and powder fluidity, which can affect their applications in food (10). SEM images of the control, SE-LDF, BM-LDF and AT-LDF were shown in Figure 1. SEM images of all samples showed significantly different, except that there were some irregular clusters and spherical substances on the surface of samples, which were most likely the results of adsorbed small particle size fibers or residual bioactive compounds such as proteins and flavonoids (12).

The SEM of control showed that the fiber texture was clear and complete, and the surface was relatively flat and smooth without obvious holes and crakes. The surface of SE-LDF had a pronounced dendritic fibrous structure and few holes. This might be due to fluid shear stress and liquid layer friction forces of SE promoting particle breakage (9) and large porosity (10, 12), leading to more loose binding between components. With the removal of other components around the fiber bundle under fluid turbulence, the main structure of the fiber was preserved (32). In combination with the subsequent cellulase hydrolysis treatment, this pronounced dendritic fibrous structure and holes appeared on the surface of SE-LDF. According to the SEM of BM-LDF, the fiber texture and particle size of LDF were significantly changed by BM. Compared with other samples, the particle size was the smallest and more particles were aggregated with each other. This result might be attributed to the strong high collision, shear force and friction force caused by BM, which may severely destroy the glycosidic bond of the fiber and the hydrogen bond force between the molecules, resulting in particle breakage (7). Meanwhile, low particle size and high specific surface area leaded to an increase in the adsorption capacity of particles, making the irregular clusters and spherical substances on the surface of BM-LDF more than other samples (33). The SEM of AT-LDF exhibited a honeycomb type structure with relatively large regular holes, and relatively few irregular clusters and spherical substances on the surface. It has been reported that high heat treatment and high pressure treatment in AT can make the cellulose structure loose and expand, increase the holes, and destroy peptide bonds and glycosidic bonds, leading to transform insoluble components such as protein and polysaccharides into soluble forms (8, 18). In combination with the subsequent cellulase hydrolysis treatment, these soluble materials were removed (34) to form a honeycomb type structure with large relatively regular holes.

#### 3.1.2. Fourier transform infrared spectroscopy

**Figure 2:**
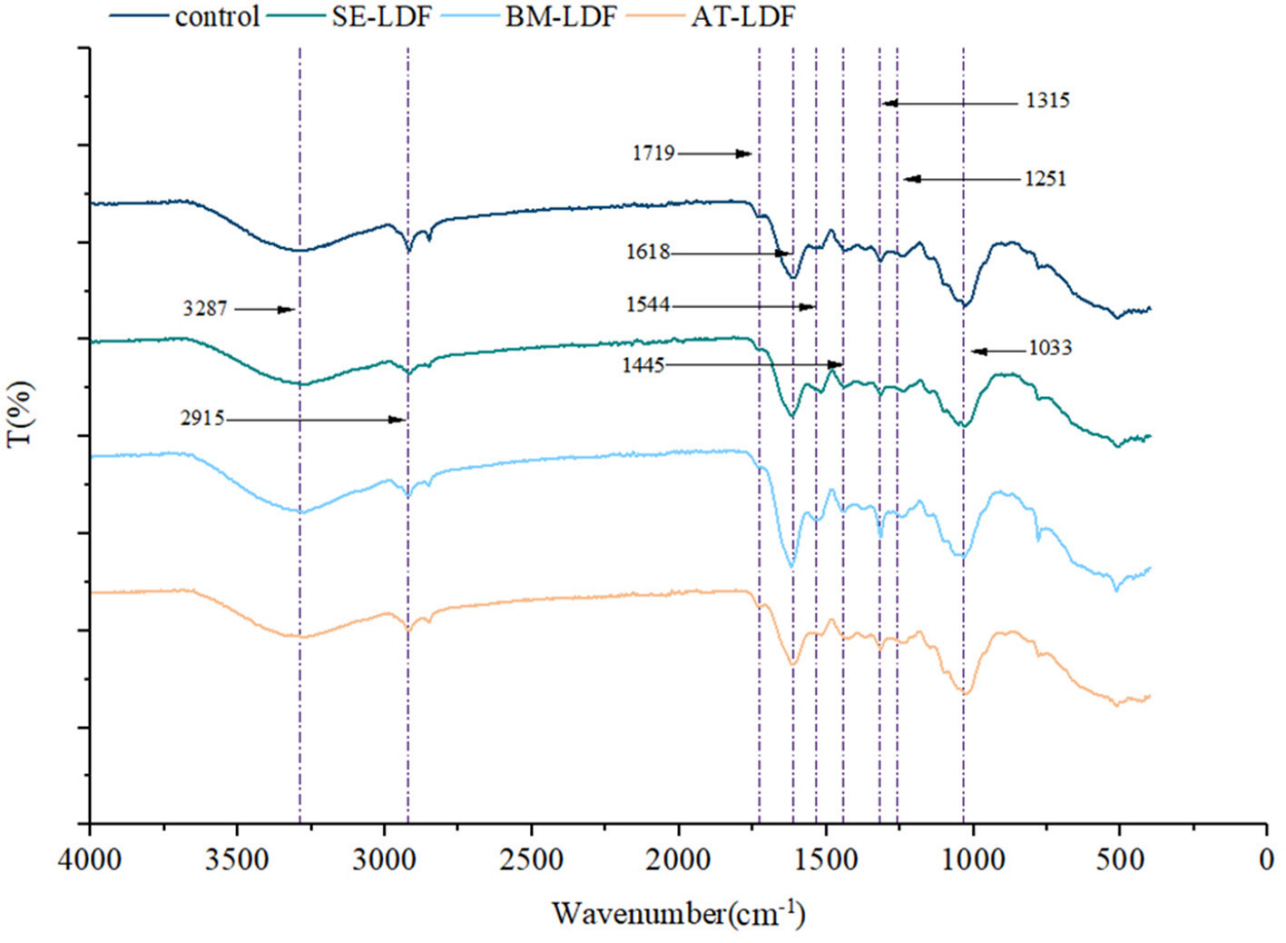
FT-IR spectra of control, SE-LDF, BM-LDF, and AT-LDF. Control: lotus leaves dietary fiber. SE-LDF, shear emulsifying modified lotus leaves dietary fiber; BM-LDF, ball milling modified lotus leaves dietary fiber; AT-LDF, autoclave modified lotus leaves dietary fiber.

FT-IR spectra of control, SE-LDF, BM-LDF and AT-LDF were shown in Figure 2. All samples had similar characteristic spectra and typical functional groups of insoluble cellulose, which were similar to defatted walnut flour (11), grape pomace (13), okara (soybean residue) (14) and ginseng IDF (35). The broad absorption at 3287 cm^−1^ was mostly attributed to O-H stretching vibration in hydroxyl groups, which might be related to free hydroxyl groups exposure in cellulose, hemicellulose, lignin and phenols (36, 37). The weak peak at 2915 cm^−1^ was a result of the C-H stretching vibrations from methyl and methylene groups, representing the typical structure of cellulose and hemicellulose polysaccharides compounds (36, 38). The minor peak at 1719 cm^−1^ was assigned to the stretching vibration of C=O in hemicellulose (35). The notable peak at 1618 cm^−1^ indicated the presence of a benzene ring in lignin, which may be related to phenolic structures (13). The weak peak at 1544 cm^−1^, along with the minor peak at 1445 cm^−1^, was the aliphatic or aromatic C-H group vibration of lignin (35). The weak peak at 1315 cm^−1^ and 1251 cm^−1^ was mainly from the typical cellulose and hemicellulose structures (35, 37). The notable absorption at 1033 cm^−1^ originated from the C-O stretching vibration of C-O-C in the pyranose ring (38).

In our study, samples had similar FT-IR spectra in general, suggesting that three modification methods did not alter the main functional groups of LDF, which were similar to those of okara (soybean residue) (14) and sea buckthorn seed meal modified by BM (15), akebia trifoliata (Thunb.) koidz. Seeds modified by SE (12) and whole grain oats modified by AT (16). While the peak intensity of BM-LDF at some absorption peaks was stronger than other samples. The particle size of BM-LDF was the lowest in all samples, and previous studies have reported that BM promoted the exposure of functional groups and facilitated the access to the corresponding groups, which may be due to sharp reduction of particle size and breakage of fiber structure by mechanical effects of BM (14, 39). In addition, the major structure and content of polyphenols in BM-LDF may be also affected FT-IR spectra (37).

#### 3.1.3. X-ray diffraction

**Figure 3:**
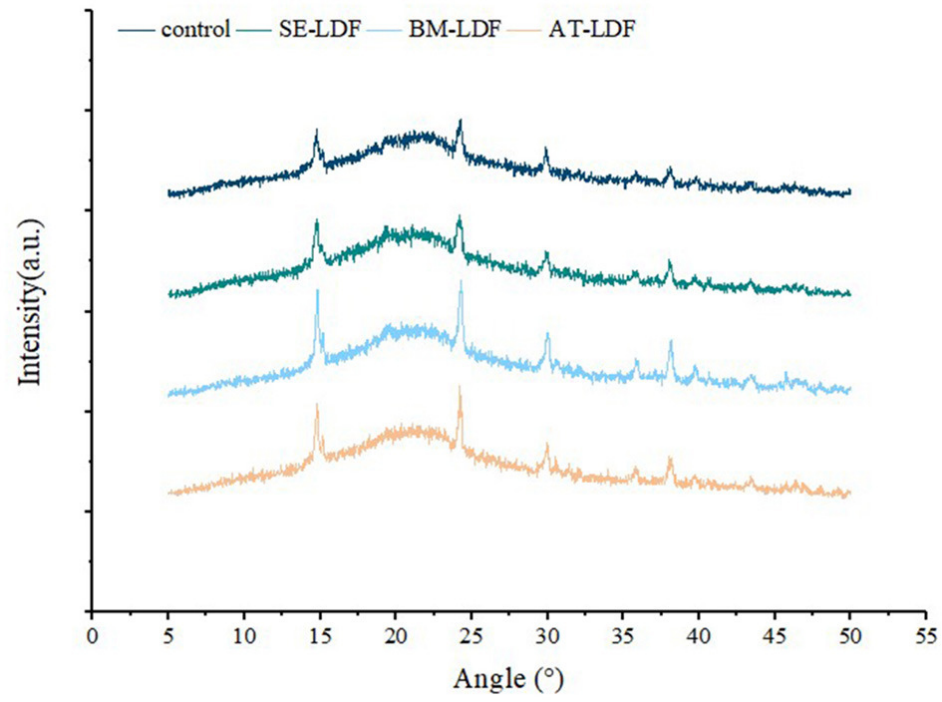
X-ray diffraction of control, SE-LDF, BM-LDF, and AT-LDF. Control: lotus leaves dietary fiber. SE-LDF, shear emulsifying modified lotus leaves dietary fiber; BM-LDF, ball milling modified lotus leaves dietary fiber; AT-LDF, autoclave modified lotus leaves dietary fiber.

Insoluble dietary fibers extracted from plant cell wall mostly contains crystalline regions due to the presence of cellulose, and amorphous regions composed of non-crystalline cellulose, hemicellulose and lignin (35, 36). X-ray diffractometry has been widely used to determine the type of crystallization structure of dietary fibers. X-ray diffraction of control, SE-LDF, BM-LDF and AT-LDF were shown in Figure 3. All samples had similar characteristic spectra, and there were characteristic diffraction peaks at a 2θ diffraction angle of 14.8° and 22.5° indicating that LDF had a crystalline cellulose I-type crystal structure (40). The three modification methods did not change the crystallization structure of LDF significantly, which was similar to those of waste orange peels modified by AT (18), deoiled cumin modified by SE (32) and okara (soybean residue) modified by BM (41). However, the intensity of some diffraction peaks changed after different modification treatments. The CrI of control, SE-LDF, BM-LDF and AT-LDF were 21.01, 15.71, 19.31 and 16.54% respectively, suggesting that the modifications leaded to the damage of the crystallization region of LDF. This result was might be attributed to modifications that reduced the CrI of LDF by destroying fiber structure, increasing fiber holes, reducing particle size and facilitating enzymatic hydrolysis (15, 32, 41). The peaks at a 2θ diffraction angle from 24° to 40° could be attributed to the denaturation of cellulose during SE, BM, AT or enzyme hydrolysis (35). Compared with other samples, SE-LDF showed the lowest CrI, suggesting SE-LDF was looser in structure and weaker at the intermolecular level. Studies have reported that low CrI was related to improving hydrophilic, lipophilic and swelling capacities of dietary fibers (15), which may be used to explain the better properties of SE-LDF in some aspects.

### 3.2. Physicochemical properties

#### 3.2.1. Dietary fiber content

SDF, IDF and TDF contents of control, SE-LDF, BM-LDF and AT-LDF were shown in Table 1. The SDF, IDF and TDF contents of control were 5.06, 72.32 and 77.38 g/100 g DW, respectively. Compared with the control, BM and AT significantly increased SDF content in LDF by 24.31 and 44.27% (*P* < 0.05), and SE decreased SDF content in LDF by 18.58% although there was no significant difference between them (*P* > 0.05). The result was similar to those of waste orange peels (18) and brewers' spent grain (8) modified by AT, and those of waste orange peels (18), citrus fiber (38) and grape pomace (13) modified by BM. This might be because the effects of AT and BM on the structure of LDF as described above, degraded the high molecular weighted fiber of AT-LDF and BM-LDF into relatively small molecular weight polysaccharides and were conducive to dissolve in the water, resulting in higher SDF contents in AT-LDF and BM-LDF than that of control (14, 18). While the strong fluid shear by SE contributed to the release of water-soluble polysaccharides (10, 12), leading to a drop in residue of water-soluble polysaccharides and a lower SDF content in SE-LDF. Compared with the control, SE, BM and AT decreased IDF content in LDF by 4.15, 6.51 and 1.56% respectively, and IDF contents in SE-LDF and BM-LDF were significantly different from that in control (*P* < 0.05). A possible explanation may be that the effects of the three modifications on the fiber structure of lotus leaves were beneficial to the degradation of cellulose in the enzymatic hydrolysis process, which reduced the IDF contents in SE-LDF, BM-LDF and AT-LDF. In general, compared with the control, AT slightly increased TDF content in LDF by 1.43% (*P* > 0.05), and SE and BM significantly decreased TDF content in LDF by 5.08% and 4.51% (*P* < 0.05).

**Table 1:** Effects of modification treatment on SDF, IDF, TDF, particle size distribution, WHC, OHC, WSC, and color of LDFs.

**Characteristics**	**control**	**SE-LDF**	**BM-LDF**	**AT-LDF**
SDF (g/100 g DW)	5.06 ± 0.43^b^	4.12 ± 0.62^b^	6.29 ± 0.49^a^	7.30 ± 0.14^a^
IDF (g/100 g DW)	72.32 ± 1.54^a^	69.32 ± 0.41^bc^	67.61 ± 1.46^c^	71.19 ± 1.01^ab^
TDF (g/100 g DW)	77.38 ± 1.49^a^	73.45 ± 0.35^b^	73.89 ± 1.68^b^	78.49 ± 0.92^a^
D_10_ (μm)	28.51 ± 0.40^a^	12.87 ± 0.11^c^	3.46 ± 0.05^d^	21.59 ± 0.33^b^
D_50_ (μm)	206.30 ± 0.95^a^	171.20 ± 0.87^b^	15.55 ± 0.35^d^	161.00 ± 0.47^c^
D_90_ (μm)	453.87 ± 4.64^a^	399.40 ± 0.65^c^	41.16 ± 0.55^d^	411.00 ± 3.83^b^
Asf(m^2^/kg)	45.89 ± 0.83^d^	74.47 ± 0.67^b^	287.67 ± 4.67^a^	61.17 ± 1.08^c^
WHC (g/g DW)	5.80 ± 0.21^b^	6.71 ± 0.21^a^	3.46 ± 0.12^d^	4.51 ± 0.12^c^
OHC (g/g DW)	3.96 ± 0.28^a^	3.73 ± 0.17^a^	2.47 ± 0.32^c^	3.18 ± 0.34^b^
WSC (mL/g DW)	7.07 ± 0.16^b^	7.92 ± 0.29^a^	5.52 ± 0.41^c^	4.84 ± 0.25^d^
L^*^	53.69 ± 0.53^b^	53.96 ± 0.56^b^	56.45 ± 0.02^a^	46.02 ± 0.76^c^
a^*^	0.85 ± 0.04^b^	0.47 ± 0.02^c^	−0.21 ± 0.01^d^	2.80 ± 0.08^a^
b^*^	13.57 ± 0.30^c^	14.41 ± 0.24^b^	20.39 ± 0.04^a^	9.48 ± 0.49^d^
ΔE	-	0.96	7.43	8.91

All values were means ± sd, n = 3. Values with different letters were significantly different in the same row, P < 0.05. “-” represented not detected. Control, lotus leaves dietary fiber; SE-LDF, shear emulsifying modified lotus leaves dietary fiber; BM-LDF, ball milling modified lotus leaves dietary fiber; AT-LDF, autoclave modified lotus leaves dietary fiber; SDF, soluble dietary fiber; IDF, insoluble dietary fiber; TDF, total dietary fiber. D_10_, D_50_ and D_90_ were equivalent volume diameters determined at 10, 50, and 90% cumulative volume, respectively. A_SF_ represented specific surface areas of powder. WHC, water holding capacity; OHC, oil holding capacity; WSC, water solubility capacity. L^*^ represented the brightness (0 is black, 100 is white); a^*^ represented the redness and greenness (a^*^ > 0, represented the degree of red; a^*^ < 0 means green degree); b^*^ represented yellow and blue degree (b^*^ > 0 means yellow degree, b^*^ < 0 means blue degree), ΔE represented color difference.

#### 3.2.2. Particle size distribution

The food added with plant dietary fibers generally presents a rough texture and bad sensory acceptance (14). Reducing the particle size of dietary fibers can improve the rough texture and relates closely to enhancing its functionality (13, 42). D_10_, D_50_ and D_90_ and specific surface areas (A_sf_) of control, SE-LDF, BM-LDF and AT-LDF were shown in Table 1. Compared with the control, SE-LDF, BM-LDF and AT-LDF showed smaller D_10_, D_50_, and D_90_ and larger A_sf_. It has been reported that high temperature, high pressure, extrusion, collision and fluid shear stress could break down glycosidic bonds and peptide bonds, which lead to particle breakage and destruction, the release of oligosaccharides and proteins, dissolution of soluble components, thus reduced particle size (16, 18). Additionally, D_10_, D_50_, and D_90_ of BM-LDF were the lowest, which were 3.46, 15.55, and 41.16 μm respectively, reaching the level of ultra-fine grinding, suggesting that the effect of BM on the particle size of LDF was stronger than that of AT and SE in our study. The BM-LDF exhibited relatively narrow particle size distribution and better particle uniformity. Many studies have also shown that BM could rapidly reduce the particle size of powder, even to the submicron level, therefore BM is commonly used in the processing of ultra-fine powder of fiber materials (3). The effects of SE and AT on the particle size of LDF were similar, and the D_50_ were 171.20 and 161.00 μm. A_sf_ of particles increases with the decrease of particle size (42), which indicated that BM-LDF may be more sufficient contact with the solution, leading to an increase in dissolving out capacity of functional components in sample and changing adsorption capacity of sample (13, 15).

#### 3.2.3. Water-holding capacity, oil-holding capacity and water swelling capacity

WHC, OHC and WSC of dietary fibers have important significance for food quality, and are influenced by many factors, such as particle size, specific surface area, porosity, crystallinity and fiber spatial structure (33, 43). WHC can retain more water in food to reduce food dehydration and contraction (44), and promote gastrointestinal peristalsis in the human body (36). OHC can make the fat in food not easy to flow out and stabilize food quality, which helps to prolong the shelf life, and accelerate fat excretion and reduce serum cholesterol levels in the human body (45). WHC affects food viscosity, and reduces intestinal transit time and delays gastric clearance speed in the human body (43). Previous studies have shown that the interaction of dietary fiber with water or oil can be summarized into two types: (i) physical forms through adsorption, encapsulation and interception of water or oil, and (ii) chemical bonds formed through the bonding of hydrophilic groups exposed to water or the bonding of lipophilic groups exposed to oil (32, 46).

WHC, OHC and WSC of control, SE-LDF, BM-LDF and AT-LDF were shown in Table 1. WHC, OHC and WSC of control were 5.80 g/g DW, 3.96 g/g DW, and 7.07 mL/g DW, respectively. WHC and WSC of SE-LDF were highest in all samples. OHC of SE-LDF was slightly lower than that of control but there was no significant difference between them (*P* > 0.05), and was significantly higher than those of BM-LDF and AT-LDF (*P* < 0.05). This result may be because fluid shear stress in SE was not excessively damaged the LDF fiber network structure and had little effect on the particle size. During the strong fluid shock, the polysaccharide, protein and other components encapsulated in LDF were more easily released so that the fiber structure of SE-LDF was looseer and the fiber gap increased, which were conducive to physical interception and the combination of chemical bonds. The result was similar to research on deoiled cumin (32), citrus peel (36) and bamboo shoot fiber (10) modified by SE. Compared with the control, BM reduced WHC, OHC, and WSC by 40.34, 37.63, and 21.92%, respectively. Ultra-fine particle size, wrinkled surface and porous structure by BM modification were beneficial to exposure of internal groups and combination of chemical bonds (15). However, some studies have shown that high-strength BM excessively damaged the network structure of dietary fiber, leading to the breakage of channels and pores, which reduced physical interception to water and oil (46). Under many factors, BM-LDF showed low WHC, OHC, and WSC. Similar observations have been reported in researches of asparagus leaf (3), grape pomace (13) and deoiled cumin (32). Compared with the control, AT reduced WHC, OHC, and WSC by 22.24, 19.70, and 31.54%, respectively. High pressure in AT destroyed integrity of fiber structure (46), and AT-LDF surface structure showed a similar honeycomb shape with large and relatively regular caves, as shown in Figure 1, which reduced steric hindrance between fiber, resulting in lower physical interception to water and oil. The result was similar to research on okara (soybean residue) (41). In general, SE was more beneficial to improve WHC, OHC, and WSC of LDF than BM and AT, which was consistent with the result of the crystallinity.

####  3.2.4. Color

**Figure 4:**
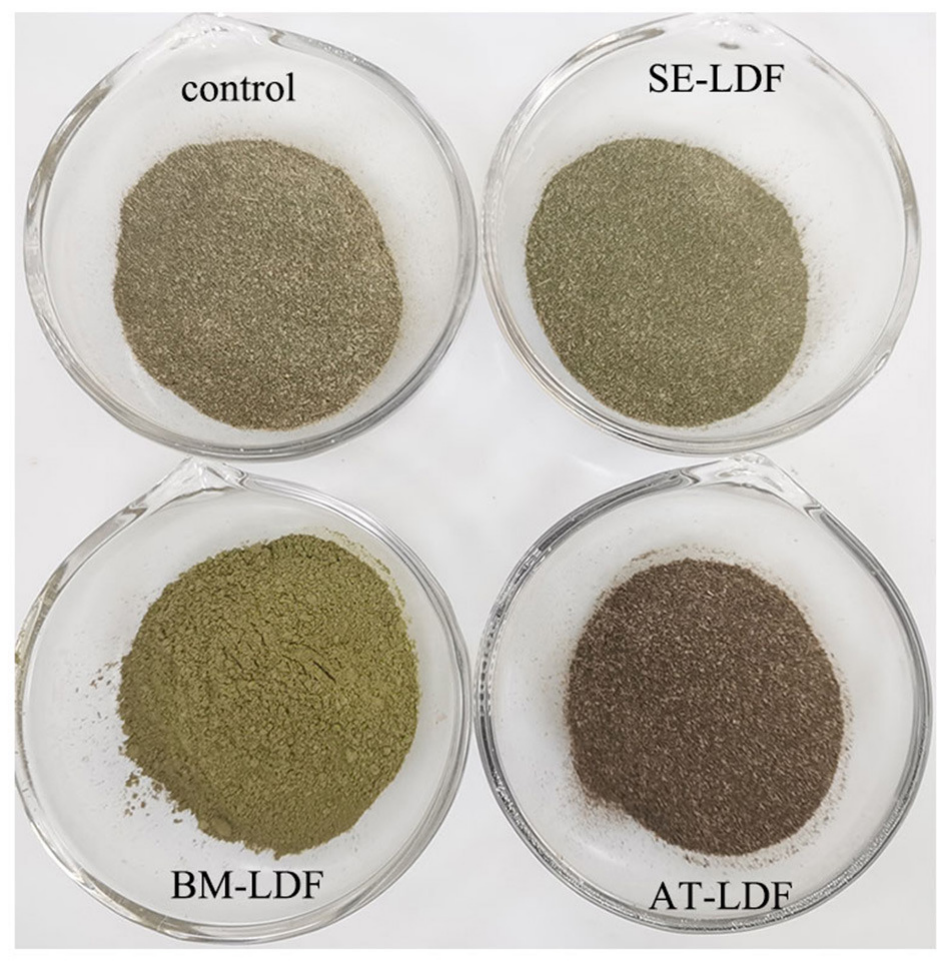
Real pictures of control, SE-LDF, BM-LDF, and AT-LDF. Control: lotus leaves dietary fiber. SE-LDF, shear emulsifying modified lotus leaves dietary fiber; BM-LDF, ball milling modified lotus leaves dietary fiber; AT-LDF, autoclave modified lotus leaves dietary fiber.

The color of raw materials in the food industry is closely related to the sensory quality of food, and can also reflect the changes in the physical structure and chemical composition of raw materials during food processing (14). Color characteristics and real pictures of control, SE-LDF, BM-LDF, and AT-LDF were shown in Table 1 and Figure 4, suggesting that modification methods significantly affected the color characteristics of LDF. The influence of modification methods on color characteristics of dietary fiber was connected with particle size, Maillard reaction, phenolic compounds aggregation, chlorophyll release and roughness of particle surface (3, 14, 17, 41). The ΔE of SE-LDF was only 0.96 compared with the control, and the effect of SE on color difference of LDF was smaller than that of BM and AT. This may be because SE had little effect on the particle size. Meanwhile, the temperature was kept constant and oxygen was isolated during SE, which effectively reduced the occurrence of related chemical reactions. The ΔE of BM-LDF was 7.43 compared with the control, and BM significantly increased the value of L^*^ and b^*^ and decreased the value of a^*^. The result was similar to the effects of BM on okara (soybean residue) (14) and asparagus leaf (3). BM-LDF was ultra-fine powder that could facilitate powder mixing and powder surface smoothing, which increased the value of L^*^. Meanwhile, particle size reduction was conducive to the exposure and aggregation of polyphenolic compounds resulting in the increase of b^*^, and was beneficial to the release of chlorophyll leading to the decrease of a^*^ (14, 41). AT-LDF had the highest ΔE of 8.91, indicating that AT showed the strongest effect on color difference. AT significantly decreased the value of L^*^ and b^*^, and increased the value of a^*^. High temperature and high pressure in AT promoted the occurrence of Maillard reaction, oxidation reaction and chlorophyll degradation, resulting in dark brown products, which increased the value of a^*^ and decreased the value of L^*^ and b^*^ (16, 41). The real pictures of samples also exhibited that BM-LDF and SE-LDF had better color quality than AT-LDF.

### 3.3. Phenolic compounds and antioxidant capacity

#### 3.3.1. Polyphenol content

**Figure 5:**
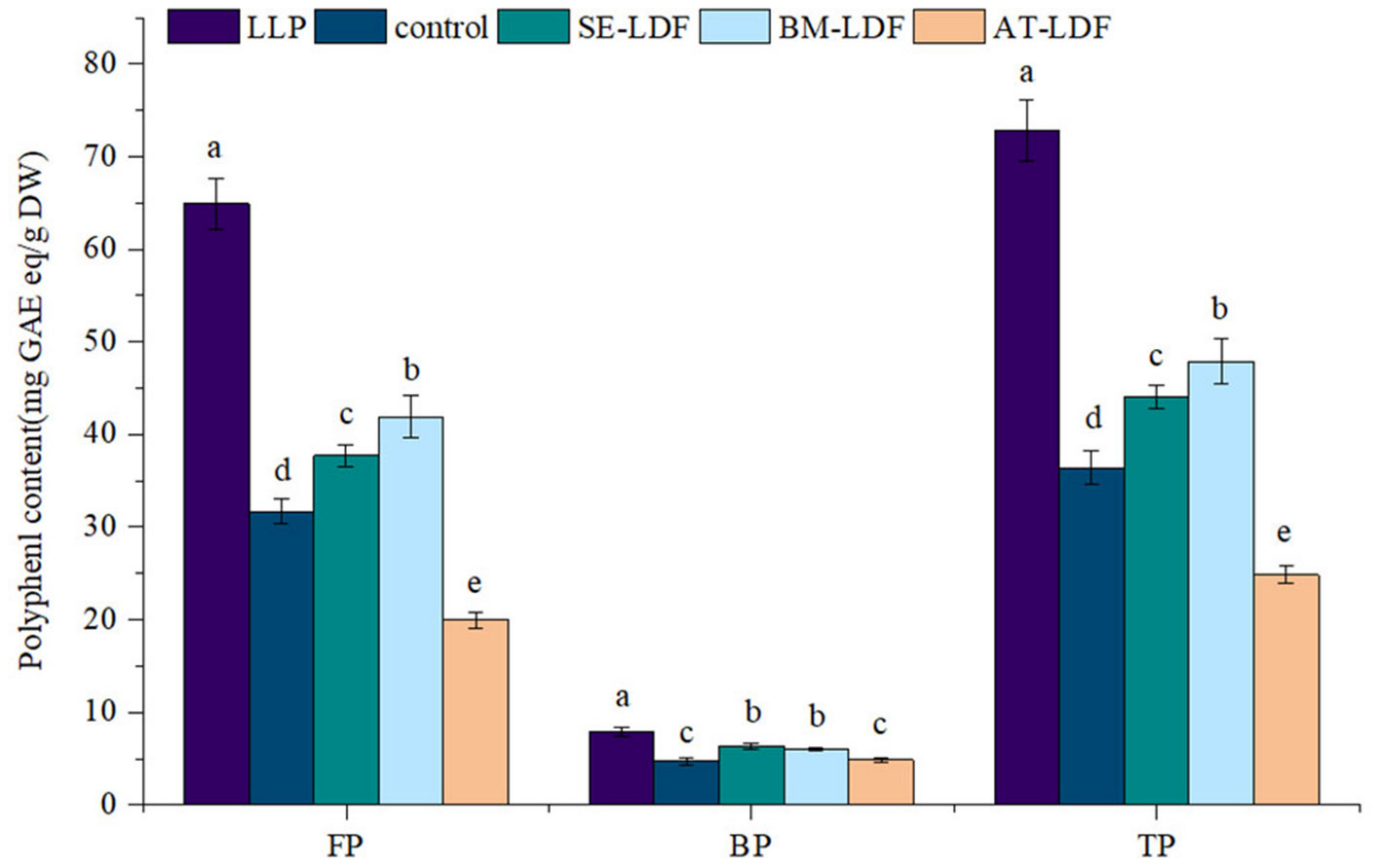
Polyphenol content of FP, BP, and TP in LLP and LDF. All values were means ± sd, *n* = 3. Values with different letters were significantly different in the same group; *P* < 0.05. FP, free phenolic; BP, bound phenolic; TP, total phenolic; LLP, lotus leaves powder; control, lotus leaves dietary fiber; SE-LDF, Shear emulsifying modified lotus leaves dietary fiber; BM-LDF, Ball milling modified lotus leaves dietary fiber; AT-LDF, Autoclave modified lotus leaves dietary fiber.

According to the different binding strengths between phenolic compounds and cell wall matrix in plants (47), phenolic compounds in plants can be divided into FP and BP, and they play different health effects on the body. The combination of FP and plant cell wall matrix is relatively weak, which can be released in the human upper digestive tract to play a healthy role (48). BP has closely bound to the plant cell wall matrix, thus can resist the digestion of the stomach and small intestine to reach the human lower gastrointestinal tract, where is released from plant cell wall matrix by colonic fermentation, and play healthy effects by producing metabolites, such as vitamins and short-chain fatty acids, forming an antioxidant environment and regulating intestinal flora (49). Lotus leaves are rich in phenolic compounds and showed a stronger antioxidative effect which is related to many factors, such as varieties, growth climate, geographic location and culturing conditions of lotus leaves (21). The FP, BP and total phenolic (TP) contents of lotus leave powder, control, SE-LDF, BM-LDF and AT-LDF were shown in Figure 5. The FP, BP, and TP contents of lotus leave powder were 64.90, 7.90, and 72.81 mg GA eq/g DW, respectively. Some phenolic components of lotus leaves were still retained in LDF extracted, and the retention rates of FP, BP and TP of lotus leaves powder were 30.74–64.53, 61.65–80.25, and 34.09–65.77%. The FP content of samples was 4.09–8.21 times that of their BP content, showing the phenolic components in lotus leaves were mainly FP, which was similar to date (Phoenix dactylifera L.) seeds (27) and rosa roxburghii tratt leaves (50).

The FP, BP and TP contents of control were 31.68, 4.81, and 37.11 mg GA eq/g DW, respectively. Compared with the control, BM and SE significantly increased FP, BP, and TP contents (*P* < 0.05). The FP and TP contents in BM-LDF were the highest, increasing by 32.20 and 29.05% than that of the control, and were significantly higher than those in SE-LDF (*P* < 0.05). The BP content in SE-LDF was the highest with an increase of 31.81% than that of the control, which was slightly higher than that in BM-LDF, but there was no significant difference between them (*P* > 0.05). Compared with the control, AT significantly decreased FP and TP contents of LDF by 37.03 and 33.12% (*P* < 0.05), and slightly increased BP content of LDF by 3.33% while there was no significant difference between them (*P* > 0.05). Overall, AT seriously damaged phenolic compounds of LDF. Studies have shown that the higher temperature could degrade phenolic compounds by promoting enzymatic oxidation and non-enzymatic oxidation (51). Some fiber modification methods, such as BM (3, 13, 14), SE (12) and AT (17), were beneficial to the release of polyphenols during extraction by destroying fiber structure, increasing fiber pores and reducing particle size, resulting in reduce phenolic content remaining in extracted dietary fiber. AT modification decreased phenolic content in LDF, which was similar to the previous research (17). However, our study showed that BM and SE increased phenolic content in LDF compared with the control, which may be due to modified fiber structure and reduced particle size caused by BM and SE, especially the particle size of BM-LDF, which were conducive to the contact between LDF and solvent in the extraction of LDF polyphenols, improving the release of phenolic compounds from LDF. Overall, BM and SE increased the content of phenolic compounds in LDF compared with the control.

#### 3.3.2. Flavonoids composition

**Figure 6:**
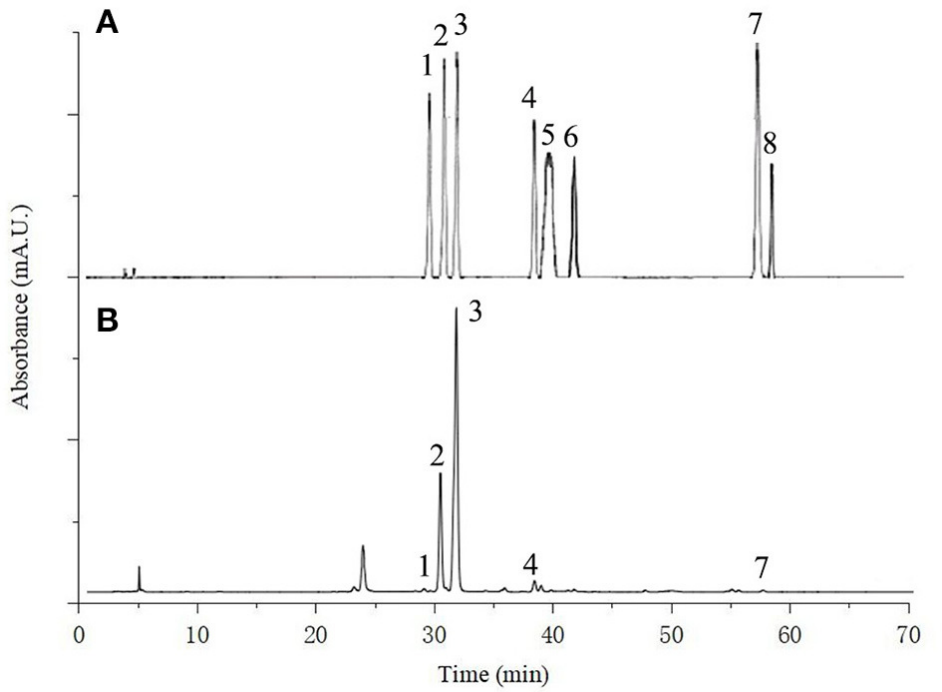
The HPLC chromatogram of **(A)** standard mixture solution of eight flavonoids [rutin (1) hyperoside (2) isoquercitrin (3) astragaline (4); catechin (5) myricetin (6) quercetin (7) and kaempferol (8)]; **(B)** free phenolic of lotus leaves powder.

In recent years, flavonoids compounds have been widely accepted as a category of the most important biologically active components of lotus leaves. Many previous studies have identified flavnoids of lotus leaves, mainly including rutin, hyperoside, catechin, quercetin, quercetin-3-O-glucopyranoside, isoquercitrin, astragalin, taxifolin, etc., and their type, content and properties related to varieties, growth climate, geographical location, culture conditions and other factors of lotus leaves (21). Figure 6 shows the chromatogram of a standard mixture solution and FP of lotus leaves powder. Five flavonoids were identified in lotus leaves powder: rutin, hyperoside, isoquercitrin, astragalin and quercetin, and catechin, myricetin and kaempferol were not detected in all samples. The result was similar to Liao et al. (52), which may be because the lotus leaves samples came from Hunan China, with similar geographical location and climatic conditions, agricultural and environmental factors. The flavonoids of FP and BP in lotus leaves powder, control, SE-LDF, BM-LDF, and AT-LDF were shown in Table 2. The result exhibited that the type of main flavonoids in FP and BP of lotus leaves powder were the same, including the above five flavonoids, and the flavonoids content of FP was higher than that of BP. Compared with lotus leaves powder, flavonoids composition of control, SE-LDF, BM-LDF, and AT-LDF decreased after enzymatic extraction.

**Table 2:** Flavonoids composition of FP and BP in LLP and LDFs (mg/100 g DW).

	**Sample**	**Rutin**	**Hyperoside**	**Isoquercitrin**	**astragalin**	**Catechin**	**Myricetin**	**Quercetin**	**Kaempferol**
FP									
	LLP	20.76 ± 3.81	710.76 ± 141.10	1998.14 ± 373.52	82.73 ± 17.98	-	-	10.91 ± 1.49	-
	Control	-	3.70 ± 0.45	724.85 ± 54.48	18.70 ± 2.11	-	-	-	-
	SE-LDF	-	-	176.35 ± 40.22	3.44 ± 0.44	-	-	-	-
	BM-LDF	-	119.03 ± 15.13	477.20 ± 45.34	9.98 ± 0.75	-	-	7.74 ± 1.78	-
	AT-LDF	4.50 ± 0.18	153.15 ± 27.52	701.51 ± 87.90	20.32 ± 3.82	-	-	-	-
BP									
	LLP	1.57 ± 0.31	62.03 ± 14.60	532.23 ± 90.48	6.10 ± 1.39	-	-	1.23 ± 0.13	-
	Control	0.41 ± 0.02	1.93 ± 0.34	148.03 ± 4.50	5.79 ± 0.08	-	-	0.77 ± 0.01	-
	SE-LDF	0.20 ± 0.06	3.00 ± 0.22	55.20 ± 6.84	1.67 ± 0.05	-	-	1.07 ± 0.10	-
	BM-LDF	-	8.28 ± 0.67	80.05 ± 5.29	1.16 ± 0.05	-	-	0.92 ± 0.03	-
	AT-LDF	0.43 ± 0.05	24.65 ± 5.36	116.27 ± 26.98	3.59 ± 0.31	-	-	-	-

All values are means ± sd, n = 3.“-” means not detected. FP, free phenolic; BP, bound phenolic; LLP, lotus leaves powder; Control, lotus leaves dietary fiber; SE-LDF, shear emulsifying modified lotus leaves dietary fiber; BM-LDF, ball milling modified lotus leaves dietary fiber; AT-LDF, autoclave modified lotus leaves dietary fiber.

Researchers have reported that the contents of isoquercitrin, hyperoside and astragalin in lotus leaves were relatively high (22, 23, 52). Modification decreased isoquercitrin content in LDF. Compared with the control, AT, BM, and SE reduced isoquercitrin of FP in LDF by 3.22, 34.17, and 75.67%, and reduced isoquercitrin of BP in LDF by 21.46, 45.92, and 62.71%. In general, modifications increased hyperoside content in LDF compared with the control. Hyperoside contents of FP and BP in AT-LDF were the highest, respectively 41.39 and 12.77 times of control. Compared with the control, AT increased astragalin of FP in LDF by 8.66%, and BM and SE reduced astragalin of FP in LDF by 46.63 and 81.60%. AT, BM and SE reduced astragalin of BP in LDF by 37.40, 79.97, and 71.16%. In addition, modification decreased rutin and quercetin content in LDF. Overall, AT-LDF showed higher flavonoids contents, and SE-LDF showed lower flavonoids contents among the three modified LDFs. This result was contrary to the polyphenol content of LDF. The phenolic compounds in lotus leaves were mainly identified as phenolic acids and flavonoids in previous research (21). So this result may be because the polyphenol extract of LDF contained phenolic acids and other polyphenol components that were not concerned in our study.

#### 3.3.3. Antioxidant capacity

**Figure 7:**
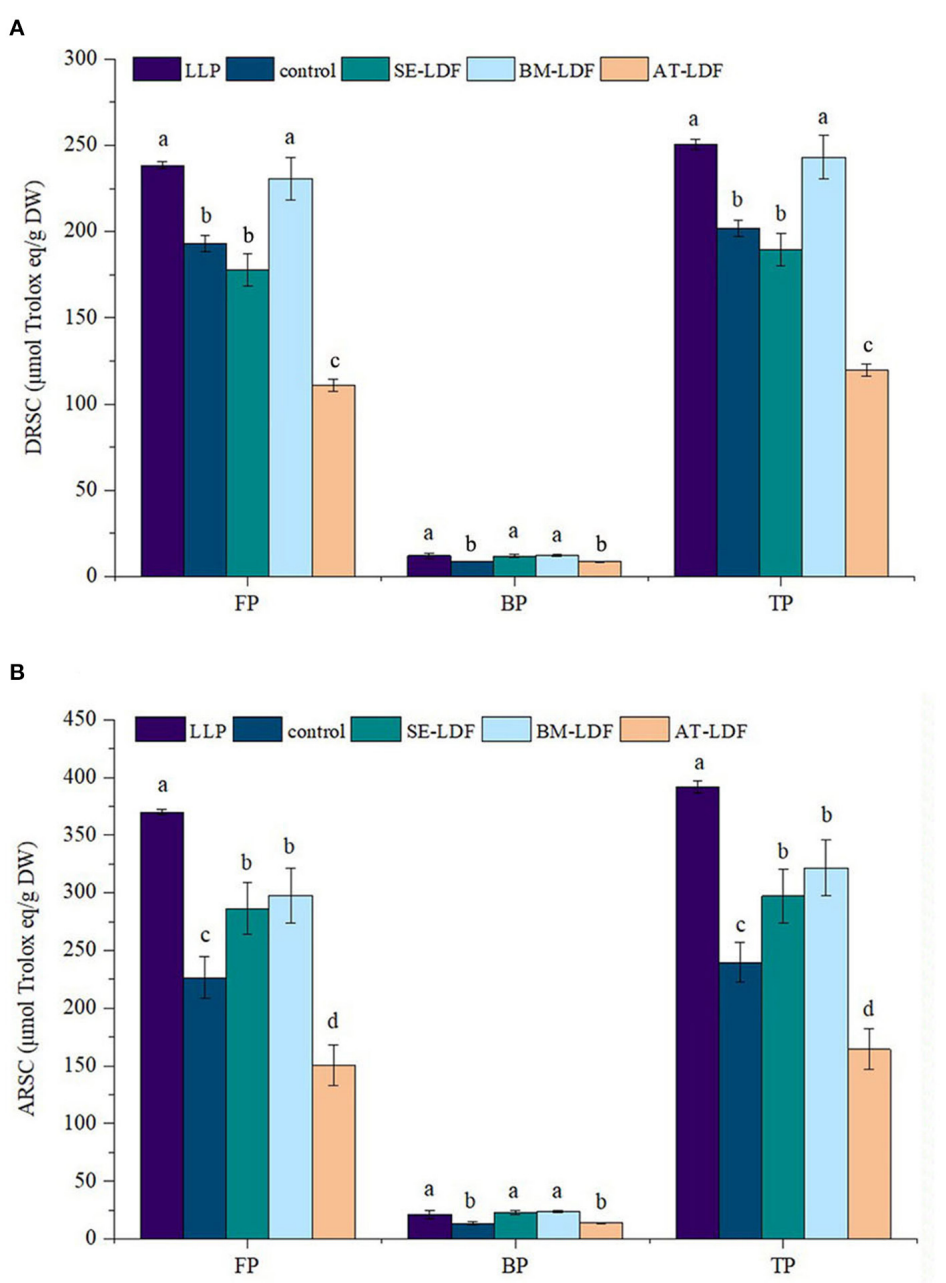
DRSC and ARSC of FP, BP, and TP in LLP and LDFs. **(A)** DRSC, DPPH free radical scavenging capacity; **(B)** ARSC, ABTS free radical scavenging capacity. All values were means ± sd, *n* = 3. Values with different letters were significantly different in the same group, *P* < 0.05. FP, free phenolic; BP, bound phenolic; TP, total phenolic. LLP, lotus leaves powder; control, lotus leaves dietary fiber; SE-LDF, shear emulsifying modified lotus leaves dietary fiber; BM-LDF, ball milling modified lotus leaves dietary fiber; AT-LDF, autoclave modified lotus leaves dietary fiber.

The DRSC and ARSC are simple, economical and effective methods to evaluate the antioxidant properties of food, which have been widely used in the researches of functional foods. DRSC and ARSC of lotus leaves powder, control, SE-LDF, BM-LDF and AT-LDF were shown in Figure 7. The FP DRSC, BP DRSC and TP DRSC of lotus leaves powder were 238.48, 12.12 and 250.60 μmol Trolox eq /g DW, respectively. Compared with lotus leaves powder, DRSC of control, SE-LDF, BM-LDF and AT-LDF decreased after enzymatic extraction, while there was no significant difference in DRSC between lotus leaves powder and BM-LDF. The FP DRSC, BP DRSC and TP DRSC of control were 193.01, 8.84, and 201.85 μmol Trolox eq /g DW, respectively. BM-LDF showed the higher FP DRSC, BP DRSC and TP DRSC, increasing by 19.56, 38.80, and 20.40% than those of control, and there were significant differences with other samples (*P* < 0.05). SE showed significantly higher BP DRSC than that of control, increasing by 35.63% (*P* < 0.05), and slightly decreased FP DRSC and TP DRSC by 7.95 and 6.04% than those of the control. AT significantly decreased FP DRSC and TP DRSC by 42.47 and 40.66% than those of control (*P* < 0.05), and slightly decreased BP DRSC by 1.13% while there was no significant difference between them (*P* > 0.05).

The FP ARSC, BP ARSC and TP ARSC of lotus leaves powder were 369.95, 21.36, and 391.31 μmol Trolox eq/g DW, respectively. Compared with lotus leaves powder, FP ARSC and TP ARSC of control, SE-LDF, BM-LDF, and AT-LDF were significantly decreased after enzymatic extraction (*P* < 0.05). The FP ARSC, BP ARSC, and TP ARSC of control were 226.42.06, 13.39, and 239.81 μmol Trolox eq/g DW, respectively. The ARSC of SE-LDF and BM-LDF were significantly higher than those of control. The ARSC of BM-LDF was slightly higher than that of SE-LDF, and there was no significant difference between them (*P* > 0.05). Similar to the effect of AT on DRSC, AT significantly decreased FP ARSC and TP ARSC by 33.54 and 31.45% than those of the control (*P* < 0.05), and slightly increased BP ARSC by 3.88% while there was no significant difference between them (*P* > 0.05).

Those data showed a positive correlation between antioxidant activity and polyphenol content, which was similar to previous studies (39, 50). In general, DRSC and ARSC of FP were significantly higher than that of BP in LDF, and DRSC and ARSC of BM-LDF and SE-LDF were higher than those of the control and AT-LDF, suggesting BM and SE were contributed to the antioxidant activity of LDF.

## 4. Conclusion

Lotus (*Nelumb*o) leaves fiber exhibit a dense structure and poor taste. In this study, the three physical modifications of SE, BM, and AT were utilized to improve the properties of LDF. The results showed that the three modifications could change the microstructure of LDF and reduced the CrI of LDF to obtain a looser structure, and affected the physicochemical properties, polyphenol content, flavonoids composition and antioxidant capacity *in vitro* of LDF. In general, SE contributed to the looser structure and better physical characteristics of LDF, which may be more conducive to LDF promoting gastrointestinal peristalsis by water swelling, and reducing high-calorie components absorption by physical interception. SE-LDF may be more applicable as a functional food raw material with low caloric requirements. BM effectively reduced the particle size to improve the taste, facilitated the dissolution of bioactive components and increased the antioxidant capacity of LDF to play the functional characteristics. BM-LDF may be more applicable as a functional food raw material with better antioxidant function. Although AT improved the dense structure of LDF, it showed adverse effects on the physical properties, phenolic compounds and the antioxidant capacity of LDF. Overall, SE and BM are appropriate modifications to enhance the properties of LDF with their own advantages, and researchers should select appropriate modifications of LDF based on exploitation requirements.

## Data availability statement

**T**he original contributions presented in the study are included in the article/supplementary material, further inquiries can be directed to the corresponding authors.

## Author contributions

HZ designed the research content, performed the experiments, analyzed the data, and wrote the manuscript. YS performed the experiments, analyzed the data, and wrote the manuscript. TZhe and YZ analyzed and discussed the data. LF and TZho performed the experiments and analyzed the data. FJ and YX wrote the manuscript. YY and KH designed the research content, analyzed the data, and modified the manuscript. All authors read and approved the final manuscript.
